# Prediction of venous thromboembolism with machine learning techniques in young-middle-aged inpatients

**DOI:** 10.1038/s41598-021-92287-9

**Published:** 2021-06-18

**Authors:** Hua Liu, Hua Yuan, Yongmei Wang, Weiwei Huang, Hui Xue, Xiuying Zhang

**Affiliations:** 1 China-Japan Union Hospital of Jilin University, Changchun, Jilin 130000 People’s Republic of China; 2 School of Nursing, Jilin University, Changchun, 130021 Jilin People’s Republic of China; 3 The Second Hospital of Jilin University, Changchun, 130000 Jilin People’s Republic of China; 4 Department of Histology & Embryology, College of Basic Medical Sciences, Jilin University, Changchun, 130021 People’s Republic of China

**Keywords:** Diseases, Health care, Risk factors

## Abstract

Accumulating studies appear to suggest that the risk factors for venous thromboembolism (VTE) among young-middle-aged inpatients are different from those among elderly people. Therefore, the current prediction models for VTE are not applicable to young-middle-aged inpatients. The aim of this study was to develop and externally validate a new prediction model for young-middle-aged people using machine learning methods. The clinical data sets linked with 167 inpatients with deep venous thrombosis (DVT) and/or pulmonary embolism (PE) and 406 patients without DVT or PE were compared and analysed with machine learning techniques. Five algorithms, including logistic regression, decision tree, feed-forward neural network, support vector machine, and random forest, were used for training and preparing the models. The support vector machine model had the best performance, with AUC values of 0.806–0.944 for 95% CI, 59% sensitivity and 99% specificity, and an accuracy of 87%. Although different top predictors of adverse outcomes appeared in the different models, life-threatening illness, fibrinogen, RBCs, and PT appeared to be more consistently featured by the different models as top predictors of adverse outcomes. Clinical data sets of young and middle-aged inpatients can be used to accurately predict the risk of VTE with a support vector machine model.

## Introduction

Venous thromboembolism (VTE) is the third most common cause of death and the leading cause of sudden death in hospitalized medical patients^1^. VTE includes deep venous thrombosis (DVT) and pulmonary embolism (PE). DVT is a dominant risk factor for PE. Many studies have shown that the incidence of PE has gradually increased in recent years^1–4^. It is remarkable that a considerable proportion of these patients with DVT are asymptomatic^5,6^, which has resulted in considerable difficulties for clinicians rescuing these patients. Prophylaxis is widely recognized as an effective method for reducing VTE in hospitalized patients^7^. However, the administration of VTE prophylaxis in these patients is still underused because of the lack of accurate and individual assessment of VTE risk.

A previous study confirmed that advanced age is the strongest determinant and most prevalent risk factor for VTE events^8^. Although VTE is mainly a disease of older age, and is rare before late adolescence^9^, it is still an important problem for young and middle-aged inpatients. Hospitalization significantly increases the risk of VTE in young and middle-aged people. In the United States, approximately 50% of all VTE events are due to current or recent hospital admission; almost all hospitalized patients have ≥ 1 VTE risk factor; and approximately 40% of patients have ≥ 3 VTE risk factors^10^. Beatriz found through the analysis of data obtained from a large registry of consecutive patients with VTE that one in every 50 such patients was aged 10–24 years^11^. The incidence of VTE in young and middle-aged people has also increased gradually^12^. Thus, the assessment of VTE risk for young and middle-aged inpatients should not be underestimated^13^.

Linnemann’s study demonstrated that the risk factors for VTE among people aged 20–39 years are different from those among elderly people^14^. In addition, risk factors for recurrent venous thromboembolism in young and middle-aged women are different from those in elderly people^15^. Beatriz found that 97% of young PE patients were at low risk according to their PESI score, and 90% were at very low risk^11^. These observations prompted us to draw attention to the risk factors for VTE among young and middle-aged people. However, a risk assessment model (RAM) lacks accurate and individual assessments of VTE risk for young-middle-aged people (≤ 45 years). Thus, predicting the risk of VTE in an individual preferring to young-middle-aged inpatients alone is necessary.

The aim of this study was to develop a new prediction model for young-middle-aged people using machine learning methods. Currently, several machine learning methods can be applied to clinical data sets. This study compared and analysed the predicted results from five models. Absolute predicted risks of VTE were generated on the basis of young-middle-aged people’s individualized clinical risk profile and could be helpful for care providers in guiding the management and prevention of VTE in young-middle-aged hospitalized patients.

## Results

### Patient characteristics and outcomes

**Figure 1:**
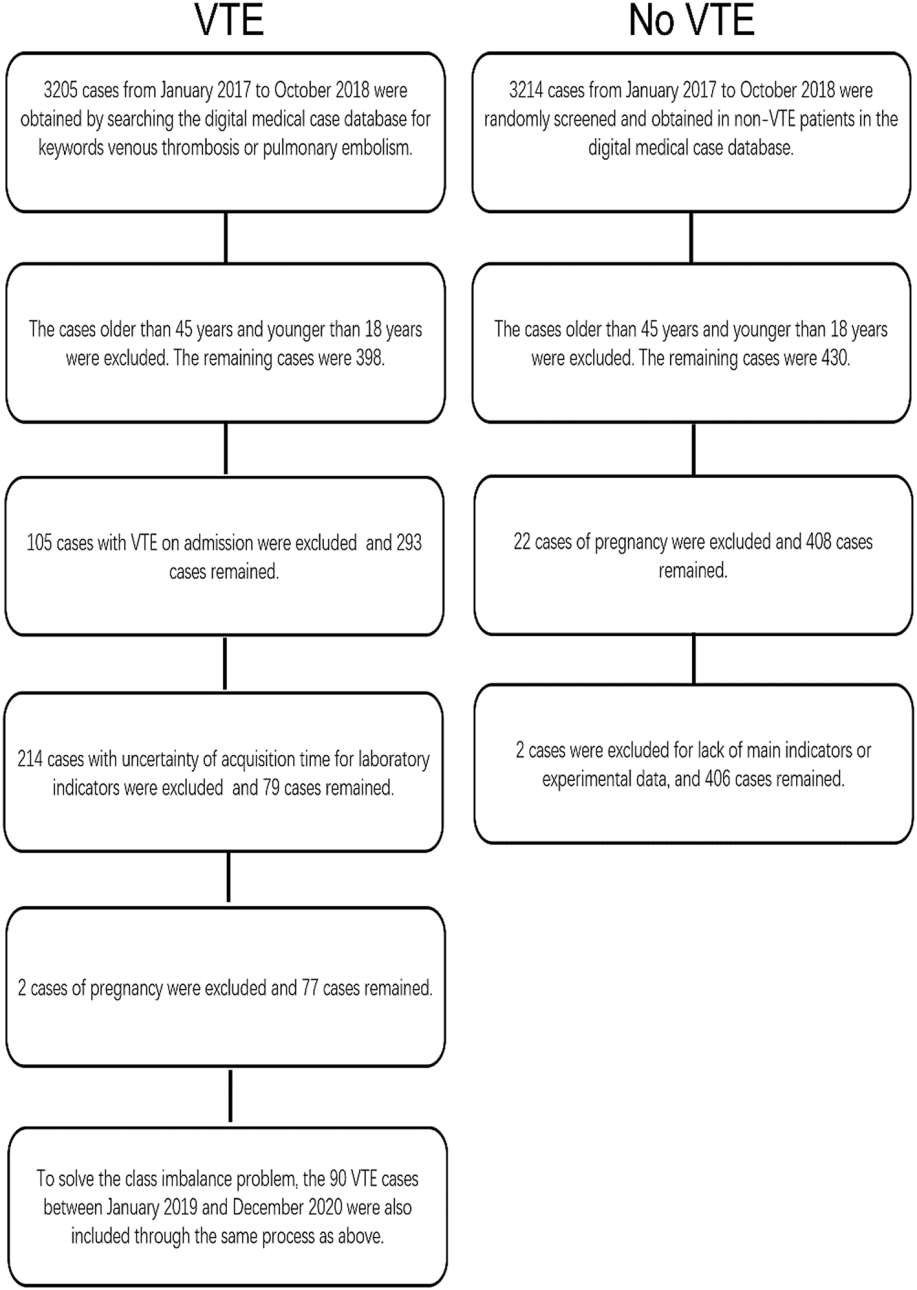
Study flow chart diagram.

**Figure 2:**
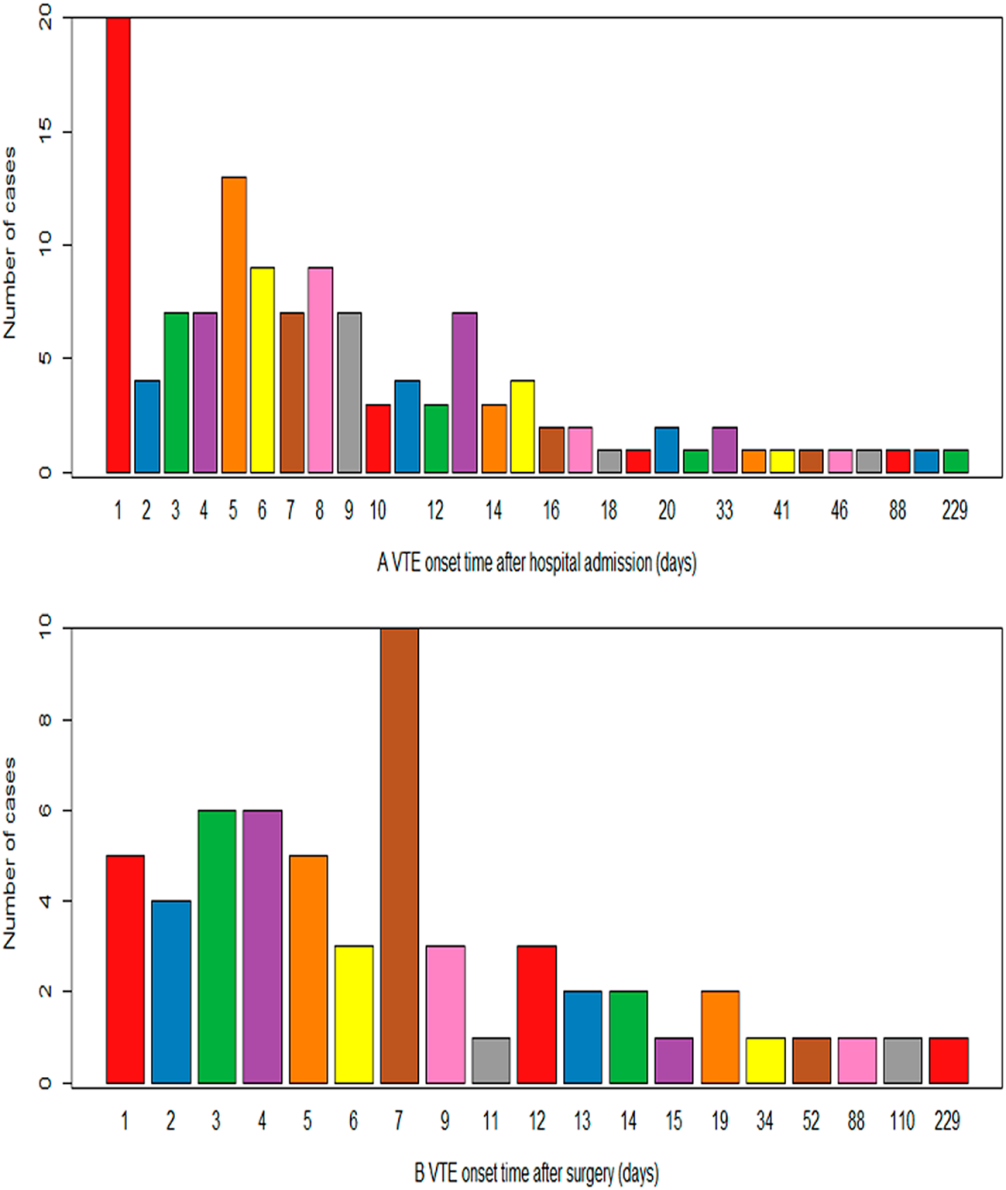
The VTE onset time distribution.

Patient characteristics and outcomes are shown in Table 1. Of 573 patients who were residents, 167 developed symptomatic, image-confirmed DVT and/or PE, and 406 patients without DVT or PE were involved in the study (Fig. 1). Patients who developed DVT and/or PE were similar to those who did not with respect to the age composition of the population and BMI. The number of male patients in the DVT and/or PE group was significantly higher than that in the non-DVT and/or PE group (P < 0.05). In addition, differences in comorbidities between the two groups based on bivariate, unadjusted comparisons were noted. For example, patients with DVT and/or PE more often had life-threatening illness (P < 0.01) and paraplegia (P < 0.01). A history of prior DVT (P < 0.01), history of prior PE (P < 0.05), history of any VTE event (P < 0.05), CVC or PICC insertion (P < 0.01), and prophylactic treatment (P < 0.01) were also more common in patients with DVT and/or PE than in those without DVT and/or PE. With respect to blood biochemical examination, significant differences between the patients with and without DVT and/or PE were also observed. The VTE onset time distribution is shown in Fig. 2.

**Table 1:** General characteristics of patients with and without DVT or PE (n = 573).

Category/variable	Modifier	No DVT and/or PE (n = 406)	Confirmed DVT and/or PE (n = 167)	P
**Patient characteristics**				
Male gender		199 (49.0%)	102 (61.1%)	0.011
Age group	< 20	22 (5.4%)	2 (1.2%)	0.039
	20–29	72 (17.7%)	23 (13.8%)	0.300
	30–39	135 (33.3%)	73 (43.7%)	0.023
	40–45	177 (43.6%)	69 (41.3%)	0.683
BMI	Median (IQR)	23.5 (4.8)	24.6 (5.4)	0.056
Hypertension		48 (11.8%) (missing value = 0)	21 (12.6%) (missing value = 0)	0.912
Myocardial infarction		0 (missing value = 0)	1 (0.6%) (missing value = 0)	0.646
Peripheral vascular disorders		5 (1.2%) (missing value = 0)	2 (1.2%) (missing value = 0)	0.700
Cerebrovascular disease		22 (5.4%) (missing value = 0)	12 (7.2%) (missing value = 0)	0.536
Active inflammation		73 (17.9%) (missing value = 0)	28 (16.8%) (missing value = 0)	0.821
Rheumatoid disease		4 (1.0%) (missing value = 0)	2 (1.2%) (missing value = 0)	0.822
Immune system diseases		4 (1.0%) (missing value = 0)	0 (missing value = 0)	0.462
Digestive tract ulcer		7 (1.7%) (missing value = 0)	0 (missing value = 0)	0.197
Diabetes without complications		18 (4.4%) (missing value = 0)	4 (2.4%) (missing value = 0)	0.360
Diabetes with complications		2 (0.5%) (missing value = 0)	2 (1.2%) (missing value = 0)	0.712
Renal disease		24 (5.9%) (missing value = 0)	10 (6.0%) (missing value = 0)	0.874
Hemi- or paraplegia		4 (1.0%) (missing value = 0)	14 (8.4%) (missing value = 0)	1.361E-05
Mild liver disease		17 (4.2%) (missing value = 0)	8 (4.8%) (missing value = 0)	0.923
Moderate to severe liver disease		11 (2.7%) (missing value = 0)	7 (4.2%) (missing value = 0)	0.509
Active cancer		58 (14.3%) (missing value = 0)	16 (9.5%) (missing value = 0)	0.165
History of DVT (Within 30-day history)		1 (0.2%) (missing value = 0)	8 (4.8%) (missing value = 0)	0.000
History of PE (Within 30-day history)		0 (missing value = 0)	3 (1.8%) (missing value = 0)	0.038
History of any VTE event (Within 30-day history)		0 (missing value = 0)	3 (1.8%) (missing value = 0)	0.038
Life-threatening illness		1 (0.2%) (missing value = 0)	48 (28.7%) (missing value = 0)	9.221E−28
History of prior CVA/TIA		3 (0.7%) (missing value = 0)	1 (0.6%) (missing value = 0)	0.712
CVC or PICC insertion		0 (missing value = 0)	11 (6.6%) (missing value = 0)	1.025E−06
Surgery type	Open surgery	165 (40.5%) (missing value = 0)	69 (41.3%) (missing value = 0)	0.955
	Laparoscopic surgery	42 (10.3%) (missing value = 0)	8 (4.8%) (missing value = 0)	0.048
	Minor surgery	38 (9.3%) (missing value = 0)	5 (3.0%) (missing value = 0)	0.014
Prophylactic treatment		3 (0.7%) (missing value = 0)	16 (9.5%) (missing value = 0)	3.137E−07
Hemostatic treatment		50 (12.3%) (missing value = 0)	16 (9.5%) (missing value = 0)	0.431
Triglyceride	High (≥ 2.26 mmol/L)	50 (12.3%) (missing value = 0)	25 (15.0%) (missing value = 0)	0.472
	Mild (1.70−2.25 mmol/L)	22 (5.4%) (missing value = 0)	7 (4.2%) (missing value = 0)	0.690
	Normal (0.45 –1.69 mmol/L)	334 (82.3%) (missing value = 0)	135 (80.8%) (missing value = 0)	0.777
Total cholesterol	High (≥ 6.46 mmol/L)	11 (2.7%) (missing value = 0)	12 (7.2%) (missing value = 0)	0.025
	Mild (5.18–6.45 mmol/L)	39 (9.6%) (missing value = 0)	15 (9.0%) (missing value = 0)	0.940
	Normal (0.83–5.17 mmol/L)	356 (87.7%) (missing value = 0)	140 (83.8%) (missing value = 0)	0.274
CRP	High (≥ 5 mg/L)	18 (4.4%) (missing value = 0)	23 (13.8%) (missing value = 0)	0.000
	Normal (< 5 mg/L)	388 (95.6%) (missing value = 0)	144 (86.2%) (missing value = 0)	0.000
APTT	s	33.2 ± 5.3 (missing value = 40)	34.8 ± 7.9 (missing value = 7)	0.025
PT	s	12.8 ± 7.8 (missing value = 40)	14.4 ± 4.5 (missing value = 7)	0.003
Fibrinogen	g/L	3.2 ± 1.1 (missing value = 40)	3.9 ± 1.4 (missing value = 7)	4.081E−09
WBC	*10^9^/L	8.3 ± 4.4 (missing value = 1)	10.2 ± 5.5 (missing value = 2)	0.000
RBC	*10^12^/L	4.5 ± 0.8 (missing value = 1)	4.2 ± 0.9 (missing value = 2)	0.000
Hemoglobin	g/L	133.7 ± 28.7 (missing value = 2)	123.3 ± 28.7 (missing value = 2)	0.000
Platelet	*10^9^/L	253.7 ± 100.6 (missing value = 2)	264.7 ± 164.2 (missing value = 2)	0.489

*BMI* body mass index, *DVT* deep venous thrombosis, *PE* pulmonary embolism, *VTE* venous thromboembolism, *CVA* cerebrovascular accident, *TIA* transient ischemic attack, *CVC* central venous catheter, *PICC* peripherally inserted central venous catheters, *CRP* C-reactive protein, *APTT* activated partial thromboplastin time, *PT* prothrombin time, *WBC* white blood cell count, *RBC* red blood cell count.

### The selection of the best model

**Figure 3:**
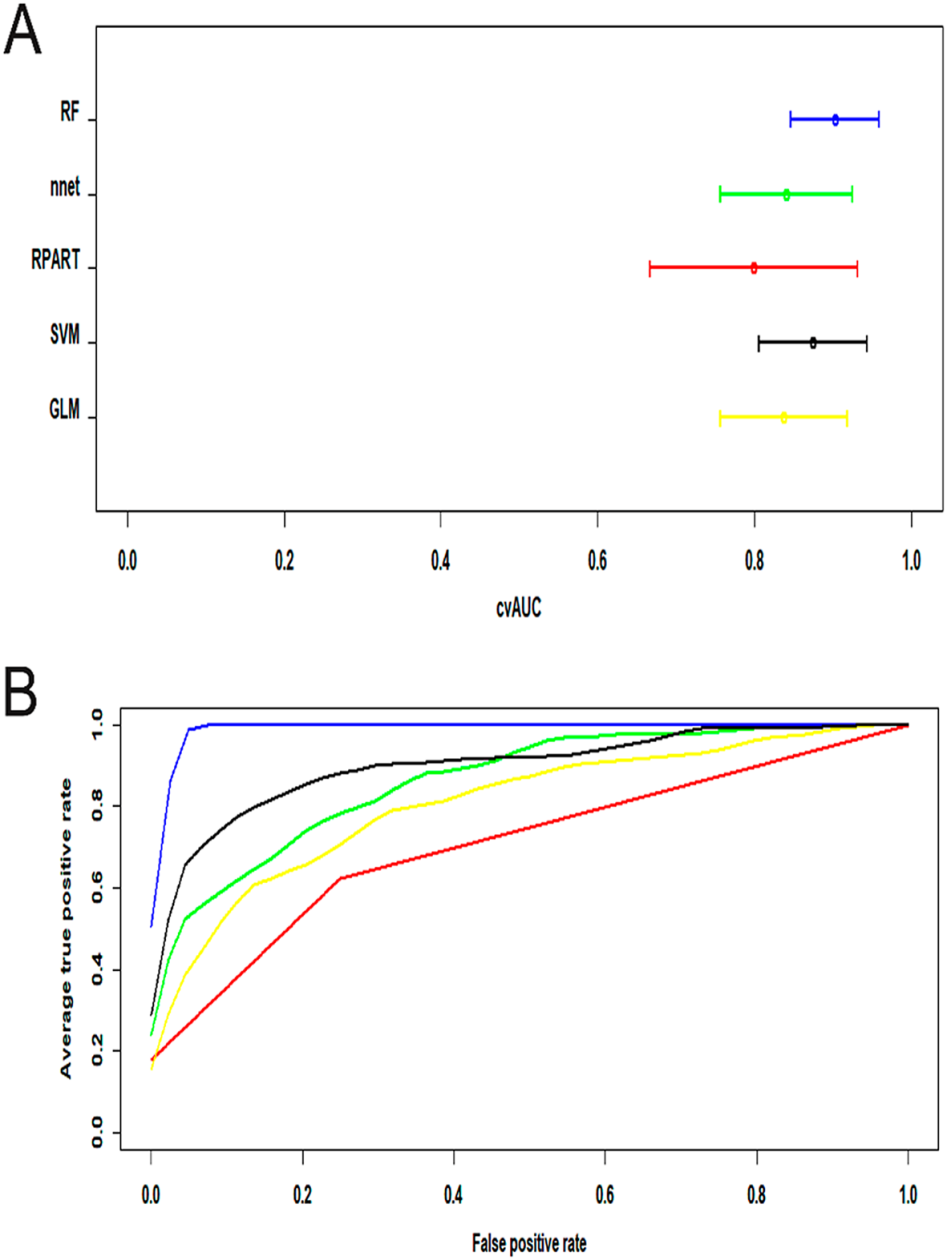
Model performance. (**A**) The cross-validated area under the receiver operator characteristic (ROC) curve (cvAUC) generated with different models with estimated 95% confidence intervals. (**B**) Consensus ROC curves generated with different models. Yellow is generalized linear, black the support vector machine, red the decision tree, green the neural network, and blue the random forest model. *GLM* generalized linear method, *SVM* support vector machine, *nnet* feed-forward neural network, *RF* random forest.

The results of the training and testing subsets are shown in Table 2. The results of the training subsets agreed well with those of the testing subsets in the support vector machine (SVM) and feed-forward neural network (nnet) models. Slight underfitting appeared in the generalized linear method (GLM) and decision tree (RPART) model, while overfitting appeared in the random forest (RF) model. The cross-validated area under the receiver operator characteristic (ROC) curve (cvAUC) generated with different models with estimated 95% confidence intervals in the testing subsets is shown in Fig. 3A, and consensus ROC curves in the testing subsets generated with different models are shown in Fig. 3B. Representative confusion matrices are shown in Table 3. It was clear that all methods except for the decision tree (RPART) yielded very similar consensus ROC areas. The SVM model achieved stable and good performance for both evaluation methods with AUC values of 0.806–0.944 for 95% CI, 59% sensitivity and 99% specificity, and 87% accuracy.

**Table 2:** cvAUC achieved with training and testing sets.

Method	cvAUC (training) mean (95% CI)	cvAUC (testing) mean (95% CI)
GLM	0.810 (0.765–0.856)	0.837 (0.756–0.919)
SVM	0.904 (0.870–0.940)	0.875 (0.806–0.944)
RPART	0.752 (0.660–0.845)	0.799 (0.667–0.931)
nnet	0.868 (0.831–0.904)	0.841 (0.756–0.925)
RF	1 (1–1)	0.850 (0.793–0.907)

Results expressed as mean (95%CI) of n = 10 trials with different seed values used to split clinical data set into training and testing subsets.

*GLM* generalized linear method, *SVM* support vector machine, *nnet* feed-forward neural network, *RF* random forest.

**Table 3:** Confusion matrices in different models.

Method	True neg (n)	False pos (n)	False neg (n)	True pos (n)	Sens (%)	Spec (%)	Acc (%)
GLM	100	1	16	25	61	99	88
SVM	100	1	17	24	59	99	87
RPART	98	3	22	19	46	97	82
nnet	98	3	18	23	56	97	85
RF	96	5	19	22	54	95	83

Results from analysis performed with the whole testing set. Sens refers to sensitivity at detecting a composite outcome (true pos/[true pos + false neg]). Spec refers to specifcity at excluding a composite outcome (true neg/[true neg + false pos]), and acc refers to the accuracy of the assignment.

*GLM* generalized linear method, *SVM* support vector machine, *nnet* feed-forward neural network, *RF* random forest, *neg* negative, *pos* positive.

### Variable rankings of the models

**Figure 4:**
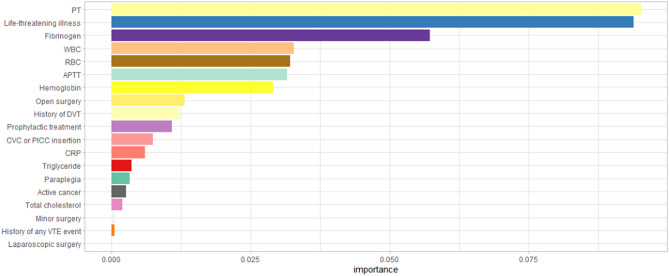
The full feature importance (SHAP value) graph of SVM model. *PT* Prothrombin time, *WBC* white blood cell count, *RBC* red blood cell count, *APTT* activated partial thromboplastin time, *DVT* deep venous thrombosis, *CVC* central venous catheter, *PICC* peripherally inserted central venous catheters; *CRP* C-reactive protein, *VTE* venous thromboembolism.

The top 4 variables in each model are shown in Table 4. Life-threatening illness, fibrinogen, RBCs, and PT appeared to be more consistently featured by the different models (≥ 3) as top predictors of adverse outcomes. In addition, these factors were considered strong predictors by both the SVM and RPART models. In particular, life-threatening illness and fibrinogen were consistently chosen as the top predictors of adverse outcomes by 4 models, and PT was selected by the SVM, RPART and RF models as having the highest importance. In addition, SHapley Additive exPlanation (SHAP) values of each feature within the SVM model are shown in Fig. 4.

**Table 4:** Top four important variables with different models.

Method	1	2	3	4
GLM	Life-threatening illness (+)	Fibrinogen (+)	Prophylactic treatment (+)	Hemoglobin (−)
SVM	PT (+)	Fibrinogen (+)	Life-threatening illness (+)	RBC (−)
RPART	PT (+)	Life-threatening illness (+)	Fibrinogen (+)	RBC (−)
nnet	Digestive tract ulcer (−)	CVC or PICC insertion (+)	History of DVT (+)	History of PE (+)
RF	PT (+)	Life-threatening illness (+)	Fibrinogen (+)	RBC (−)

*GLM* generalized linear method, *SVM* support vector machine, *nnet* neural network, *RF* random forest, *DVT* deep venous thrombosis, *RBC* red blood cell count, *APTT* activated partial thromboplastin time, *PT* prothrombin time.

+/−: the effect direction.

## Discussion

Machine learning may reduce the workload of clinicians, change diagnostic procedures, and reduce medical costs^16^. In this study, we attempted to develop a preliminary machine learning model for predicting VTE in young and middle-aged hospitalized patients. The results showed that SVM was the most accurate algorithm to predict VTE with the highest average AUC and superior statistical performance. An analysis of variables with different models showed that life-threatening illness, fibrinogen, RBCs, and PT appeared to be consistently featured by the different models as top predictors of adverse outcomes.

The goal of machine learning algorithms is to search for a linear or nonlinear function for classification or prediction^17^. Logistic regression, also known as logistic regression analysis, is a generalized linear regression analysis model^18^. The nnet is a form of supervised machine learning in which the data to be learned are neither sequential nor time-dependent^19^. RF employs decision trees to construct a predictive model on various subsamples of the dataset and uses the average value to improve the predictive accuracy and control overfitting^20^. SVM is a data classification method that involves multidimensional data sorting based on a hyperplane^21^. Decision tree (RPART), using a tree-like graph and possible consequences to classify features, is a graphic method to intuitively use probability analysis for classification or regression tasks^22^. However, the performance of machine learning algorithms varies with different data sets, and no algorithm can achieve good performance in all possible learning problems^23^. In general, the AUC value range is 0.5–1.0, with values between 0.5 and 0.7 indicating low discrimination ability, values between 0.7 and 0.9 indicating moderate discrimination ability, and values > 0.9 indicating high discrimination ability^24^. In our study, the cross-validated areas under the ROC curve were calculated to assess the accuracy of the predictive power of the models by using the cvAUC function with the tenfold cvAUC library^25^. The cvAUC values of 0.810, 0.752 and 0.868 for the training sets of GLM, RPART, and nnet, respectively, are an indication that the three models have moderate discrimination abilities. The cvAUC values of 0.904 and 1 in the training sets of SVM and RF are an indication that the two models have high discrimination ability. The generalization performance is a very important aspect in the application of machine learning algorithms. Overfitting leads to poor generalization of these models^26^. Here, overfitting occurred in the RF. Therefore, RF is not an appropriate model, although it has the best performance on the training set. In the other four models, SVM achieved the best performance (the highest cvAUC value). The confusion matrix is another widely used method to evaluate classification results^27–29^. The confusion matrix analysis found that the GLM and SVM models had the best and second-best performance (in terms of accuracy, sensitivity, and specificity), respectively. Based on the results of the two evaluation methods, SVM may be the most stable and accurate method for predicting the risk of VTE in young and middle-aged hospitalized patients.

In our study, the top predictors of adverse outcomes consistently featured by the different models were life-threatening illness, fibrinogen, RBCs, and PT (which appeared in 3 models as top predictors). Haemoglobin, prophylactic treatment, digestive tract ulcer, CVC or PICC insertion, history of DVT, and history of PE were also featured by one of these methods. Some of these risk factors have been confirmed to be related to VTE. For example, CVC or PICC insertion and a history of DVT and PE have been extensively investigated as high-risk factors for VTE^30,31^. In addition, life-threatening illness and fibrinogen have been confirmed by a recent meta-analysis to be related to the risk of VTE, and these factors are mainly related to the occurrence of VTE in the elderly^32^. However, the relationship of other factors, such as PT, haemoglobin and RBCs, has seldom been studied and is usually not associated with VTE in the elderly^32^. Recent studies confirmed that PT was an independent risk factor for prostatic tumours in the perioperative period with VTE or COVID-19-related thrombotic complications^33,34^. In addition, the relationship between red blood cells and VTE has been gradually realized^35,36^. Even more interesting is that haemoglobin has been reported to be associated with VTE risk in cancer patients^37^. However, there is no significant relationship between haemoglobin and VTE in elderly diabetic patients^38^. Our results, together with the above evidence, have provided strong support showing that the risk factors for VTE in young-middle-aged inpatients are different from those in elderly inpatients.

At present, data from VTE studies addressing the question of age-specific characteristics are scarce. Our study showed that predictors of VTE in young and middle-aged hospitalized patients were different from the risk factors included in the risk assessment model for VTE identification in hospitalized medical patients, such as the Caprini model^30^, Kucher model^39^ and Padua prediction score^40^. In the Kucher model and the Padua prediction score, elderly age was considered a high risk factor for VTE. In the Caprini model, age was subdivided into 40–60, 61–74 and 75+^30^. The study showed that the incidence of VTE strongly increases with age, which may be explained by the biology of ageing rather than by exposure to an increased number of VTE risk factors^14^. To date, we have not found any research to evaluate the effects of these models in young and middle-aged hospitalized patients. In addition, the performance of the PESI score and wells score model in predicting PE in young and middle-aged patients is poor^11,41^. Based on the different risk factors faced by patients of different ages, the above information demonstrated that it is necessary to evaluate patients of different ages separately. Therefore, the prediction model in our study will contribute to the prevention and management of VTE in young-middle-aged patients.

### Strengths and limitations

The main strength of our study was that our clinical data covered various diseases in young and middle-aged people. Additionally, the study compared and analysed the performance of five machine learning techniques for VTE. This comparison and analysis enabled a comprehensive understanding of the risk factors for VTE in young and middle-aged people and increased our confidence in our conclusions.

This study has several limitations. First, we developed the VTE model using clinical data, mainly including biochemical indicators, but did not consider other factors, such as environmental factors and genetic factors (VTE-associated genes). As a retrospective study, the selection of VTE cases and controls might result in potential selection bias^42^. Second, most of the factors included in the study were dichotomous variables rather than continuous variables, without considering the relationship between the exposure levels of these risk factors and VTE, which may hide their true relationships with VTE. Third, the risk factors predicted by different machine learning techniques are different, which caused confusion. Further study should determine the predictive value of these risk factors for VTE in young-middle-aged inpatients. Fourth, it was not possible to conduct external validation of these models due to the lack of available unique datasets at this time, so the generalization abilities of the models for other populations are still unknown.

## Conclusions

This is the first study using machine learning techniques to estimate the VTE risk for young-middle-aged inpatients. Our study confirmed that the new SVM model-predicted risk probability is helpful for care providers as it guides the management and prevention of high-risk young and middle-aged inpatients.

## Methods

### Study design and patients

The study was conducted using data for all patients who were residents of all medical departments of China-Japan Union Hospital (Jilin University, Changchun, Jilin Province, China). The data for patients who were ≤ 45 years of age and with a ≥ 2-day duration of hospitalization were included. Patients who (i) had VTE on admission, (ii) ≤ 18 years of age, (iii) were pregnant, (iv) lacked major indications and experimental data (more than 7 parameters were missing), and (v) had uncertainties in the acquisition time for laboratory indicators were excluded. Initially, data for VTE and non-VTE patients were first collected from patients between January 2017 and October 2018. Next, to solve the class imbalance problem caused by the small amount of data of patients with VTE^43^, VTE cases between January 2019 and December 2020 were also included. The study was approved by the Ethics Committee of China-Japan Union Hospital of Jilin University, Changchun, Jilin Province, China (2020081901). The research was performed in accordance with the Declaration of Helsinki. The clinical data in this manuscript were approved by the Ethics Committee of China-Japan Union Hospital of Jilin University Changchun, Jilin Province, China (2020081901). The ethics committee explicitly stated that informed consent was not required as part of this study.

### Covariates

Data on comorbidities, physical findings and laboratory and medication data were retrieved from the medical records of the hospital. Thrombosis was only recorded during hospitalization. Variables included the following: age (age ≤ 45 years), sex, hypertension, myocardial infarction, peripheral vascular diseases (vascular occlusion angeitides, Buerger disease, external jugular venous aneurysm, femoral arteriovenous fistula, popliteal artery injury, bilateral femoral artery injury, lower extremity artery injury, oesophageal and gastric varices, lower limb varicosity, lymphedema, hepatic haemangioma and intermuscular haemangioma), cerebrovascular disease (ischaemic vascular disease, haemorrhagic cerebrovascular disease and intracranial arteriovenous malformations), active inflammation (acute and chronic inflammation except for phlebitis and vasculitis), rheumatoid disease (rheumatoid arthritis, rheumatic heart disease and ankylosing spondylitis), immune system diseases (allergic dermatitis, purpura dermatosis, systemic lupus erythaematosus), digestive tract ulcer, diabetes without complications, diabetes with complications (diabetic ketoacidosis, diabetic peripheral neuropathy and diabetic ketoaciduria), renal disease, hemi- or paraplegia, mild liver disease (fatty liver, hepatic haemangioma, hepatic cyst, intrahepatic bile duct stone), moderate to severe liver disease (abnormal liver function, liver cirrhosis and hepatitis B), active cancer (admission for a cancer diagnosis or for chemotherapy), history of DVT (history of upper or lower-extremity DVT within 30 days), history of PE (within 30 days), history of any VTE event (except for the DVT and/or PE), life-threatening illness (any condition that ICU admission or transfer is required during hospitalization), history of prior CVA/TIA (cerebrovascular accident, transient ischaemic attack), CVC or PICC insertion, surgery type, prophylactic treatment, haemostatic treatment, triglyceride, total cholesterol, activated partial thromboplastin time (APTT), prothrombin time (PT), fibrinogen, white blood cell count (WBC), red blood cell count (RBC), haemoglobin, platelet, and C-reactive protein (CPR). For nonsurgical inpatients, the first laboratory index after admission was used. For hospitalized patients who underwent surgery, the laboratory index was the first laboratory examination index after the first surgery. Patients with VTE occurring before surgery were treated as nonsurgical patients. The data for variables before VTE onset were used. For categorical variables, if there was corresponding information in the medical record, they were assigned according to the corresponding information; if there was no corresponding information, they were considered normal health.

### Ascertainment of outcomes

DVT was validated based on positive compression ultrasonography and contrast venography. PE was defined based on a positive pulmonary angiogram, spiral computed tomography, and high probability ventilation/perfusion scanning.

### Statistical analyses

Analysis of 573 subjects was performed using the open-source program R (version 4.0.4)^44^. The data were cleaned by the many NAs method in the DMwR package^45^. The missing continuous data were imputed by the knnImputation method in the DMwR package with a k value of 10. Then, the subjects were randomly assigned at a ratio of 75:25 by the create Data Partition method in the CARET package^46^ into a training set (n = 431) for variable determination and model construction and a test set (n = 142) to test the model performance. The details of the variables are shown in Box 1. Five algorithms, including logistic regression, decision tree, feed-forward neural network, support vector machine, and random forest, were used for training and preparing the models.

The generalized linear method (logistic regression) model used the GLM method in the stats package^44^. A univariate logistic regression analysis was performed initially to identify significant variables (features). All significant variables with < 5% significance from univariate analysis were entered into the multiple logistic regression model using stepwise elimination to determine final variables. Other machine learning methods for decision tree, feed-forward neural network, support vector machine, and random forest models used RPART, nnet, SVM Radial, and RF methods in the CARET package, respectively. The recursive feature elimination method in the CARET package was used to identify the combination of optimal features for each machine learning model^47,48^. Tenfold cross-validation was used to minimize the overfitting or feature selection bias in the model^49–51^. To obtain the best performance of the models, the parameter cp was tuned for RPART, size and decay for nnet, sigma and C for SVM Radial, and mtry for RF.

The variables used in the GLM model included fibrinogen, haemoglobin, paraplegia, life-threatening illness, CRP, and prophylactic treatment. The variables PT, fibrinogen, life-threatening illness, RBC, haemoglobin, WBC, APTT, CRP, CVC or PICC insertion, prophylactic treatment, paraplegia, history of DVT, cholesterol, active cancer, laparoscopic surgery, minor surgery, triglyceride, open surgery, and history of any VTE event were used in the SVM model; life-threatening illness, PT, fibrinogen, RBC, haemoglobin, CVC or PICC insertion, prophylactic treatment, history of DVT, WBC, APTT, open surgery, CRP, platelet, hypertension, active inflammation, active cancer, cerebrovascular disease, cholesterol, history of prior CVA/TIA, diabetes with complications, diabetes without complications, laparoscopic surgery, haemostatic treatment, immune system diseases, mild liver disease, minor surgery, and moderate to severe liver disease were used in the RPART model; PT, life-threatening illness, fibrinogen, APTT, RBC, WBC, platelet, haemoglobin, history of DVT, CVC or PICC insertion, prophylactic treatment, cholesterol, and open surgery were used in the RF model; and haemoglobin, life-threatening illness, CVC or PICC insertion, history of DVT, digestive tract ulcer, immune system diseases, history of PE, fibrinogen, myocardial infarction, prophylactic treatment, history of any VTE event, haemostatic treatment, WBC, history of prior CVA/TIA, moderate to severe liver disease, minor surgery, and APTT were used in the nnet model.

Finally, the SVM model was constructed by using the svmRadial method with sigma = 0.1019223 and C = 0.25; the decision tree model was constructed by the rpart method with cp = 0.03571429; the RF model was constructed by the rf method with mtry = 2; and the nnet model was constructed by the nnet method with size = 1 and decay = 1e−04.

The varImp function of the CARET package was used to calculate the importance of variables in each model, and the first four variables with the highest scores were considered the top variables of the model. The full feature importance graph of the SVM model was constructed by using Scott M. Lundberg’s method^52^.

**Box 1:** Data used for predictive models.

Factors	Data type
Hypertension	Categorical
Myocardial infarction	Categorical
Peripheral vascular disorders	Categorical
Cerebrovascular disease	Categorical
Active inflammation	Categorical
Rheumatoid disease	Categorical
Immune system diseases	Categorical
Digestive tract ulcer	Categorical
Diabetes without complications	Categorical
Diabetes with complications	Categorical
Renal disease	Categorical
Hemi- or paraplegia	Categorical
Mild liver disease	Categorical
Moderate to severe liver disease	Categorical
Active cancer	Categorical
History of DVT	Categorical
History of PE	Categorical
History of any VTE event	Categorical
Life-threatening illness	Categorical
History of prior CVA/TIA	Categorical
CVC or PICC insertion	Categorical
Open surgery	Categorical
Laparoscopic surgery	Categorical
Minor surgery	Categorical
Prophylactic treatment	Categorical
Hemostatic treatment	Categorical
Triglyceride	Categorical
Total cholesterol	Categorical
C-reactive protein (CRP)	Categorical
Activated partial thromboplastin time (APTT)	Numerical
Prothrombin time (PT)	Numerical
Fibrinogen	Numerical
White blood cell count (WBC)	Numerical
Red blood cell count (RBC)	Numerical
Hemoglobin	Numerical
Platelet	Numerical

*DVT* deep venous thrombosis, *PE* pulmonary embolism, *VTE* venous thromboembolism, *CVA* cerebrovascular accident, *TIA* transient ischemic attack, *CVC* central venous catheter, *PICC* peripherally inserted central venous catheters, *CRP* C-reactive protein, *APTT* activated partial thromboplastin time, *PT* prothrombin time, *WBC* white blood cell count, *RBC* red blood cell count.

### Model comparisons

For model evaluation and validation, the cross-validated area under the receiver operator characteristic (ROC) curve (cvAUC) was determined with 10 parts in testing sets created by the create folds method in the CARET package using the method of LeDell et al.^53^. The ROC curve threshold in the calculation process was the default value of the cvAUC method in the cvAUC package^53^. The consensus ROC curve for each model was performed by using the cvAUC method in the cvAUC package. The confusion matrixes of each model in the testing sets were also used to evaluate the accuracy of the models.
